# Building Healthy Eating Knowledge and Behavior: An Evaluation of Nutrition Education in a Skill Training Course for Construction Apprentices

**DOI:** 10.3390/ijerph16234852

**Published:** 2019-12-02

**Authors:** Louisa Ming Yan Chung, Joanne Wai Yee Chung, Albert P. C. Chan

**Affiliations:** 1 Department of Health and Physical Education, The Education University of Hong Kong, 10 Lo Ping Road, Tai Po, New Territories, Hong Kong, China; joannechung@eduhk.hk; 2 Department of Building and Real Estate, Polytechnic University, 11 Yuk Choi Road, Hung Hom, Hong Kong, China; albert.chan@polyu.edu.hk

**Keywords:** nutrition education, construction apprentices, fruit and vegetable consumption

## Abstract

Background: Prior research has found poor health among construction workers is related to poor nutrition and low fruit and vegetable consumption. Promoting nutrition knowledge can improve dietary behaviors, but nutrition education among construction workers is limited. We evaluated the effectiveness of nutrition education on fruit and vegetable consumption among construction apprentices. In this pilot evaluative study, 36 construction apprentices enrolled in skill training programs received two 1.5-hour nutrition classes. Twelve questions addressing healthy eating knowledge and behavior were administered at baseline, after intervention, and at three months follow-up. After intervention, daily fruit consumption improved from baseline (mean (s.d.) =1.42 (0.55)) to post intervention (mean (s.d.) =1.72 (0.70)) (*p* < 0.05) and to three months follow-up (mean(s.d.) =1.94 (0.83)) (*p* > 0.05). After intervention, daily vegetable consumption improved from baseline (mean (s.d.) =1.67 (0.59)) to post intervention (mean (s.d.) =1.97 (0.74)) (*p* < 0.05) and to three months follow-up (mean (s.d.) = 2.19 (0.82)) (*p* > 0.05). Younger construction apprentices showed better healthy eating knowledge at post intervention and three months follow-up (*p* > 0.05). Working in normal hours showed better healthy eating knowledge at post intervention but not at three months follow up (*p* > 0.05). Both age groups and working hours did not show significant differences on healthy eating behaviour. Nutrition education implemented as a three-hour session within skill courses may possibly promote fruit and vegetable consumption among construction apprentices. Further research with control group is required to support the findings in this study.

## 1. Introduction

Noncompliance in healthy eating is a global issue, spanning different age groups, different occupations, and many countries [1,2,3,4,5,6,7,8]. Yet, this issue is of particular concern to construction workers because they are generally lower in education and faced with environmental constraints affecting healthy food choices [8,9]. Their health status also affects their productivity and immature retirement, which also affects the construction industry with insufficient labour forces. Previous surveys have found that construction workers, by occupation have low intakes of fruit and vegetables [9,10,11]. This poor eating pattern is possibly a factor contributing to construction workers having a higher risk of diet-related diseases such as obesity, diabetes, and cardiovascular diseases [12,13]. Poor health among construction workers is partly related to poor nutrition [14]. Construction workers are employed in highly risky and unfavorable work environments. First, worksites are usually located in remote and underdeveloped areas. A lack of infrastructure and temporary workplace settings make the installation of refrigeration, microwaves, storage, and preservation facilities more difficult, which prevents construction workers from preparing or storing healthy foods. The availability of facilities considerably influences the food choices of onsite construction workers [15].

In Hong Kong, the percentage of construction workers who are overweight or obese is about 50% greater than the proportion in the general adult population. The prevalence of hypertension or pre-hypertension among construction workers is 2.5 times that of adults in the region [9,12]. This alarming finding is suggested to be linked to the higher rate of mortality and morbidity among construction workers [16,17,18], which has an impact on early retirement of the workforce owing to noncommunicable diseases. Bringing a packed lunch creates a food safety issue on the work site if there are no proper means to store and reheat food. Construction workers must therefore rely on catering services located on the premises for ordering and delivering lunch boxes to their worksite at specific times. Other than these limited lunch options for construction workers, Hong Kong restaurant-style menus usually contain high levels of salt and fat. Laboratory tests conducted using the 10 most popular dishes reveal that many dishes have twice the daily limit of sodium according to recommendations of the World Health Organization [19]. In addition, long working hours and travel times from the worksite to local restaurants discourage construction workers from expanding their food options to include more healthy food choices [20]. 

Other than the limited healthy choices available around the worksites, construction workers face environmental barriers to making healthy food choices in the workplace context. However, healthy eating habits should be promoted in the construction industry to improve workers’ health, thereby reducing absenteeism and improving productivity of the workforce. As most construction workers spend more than half their waking hours performing work-related duties, previous studies suggest that workplace interventions, such as nutrition education and environmental interventions, can change workers’ dietary behaviors [21,22,23]. Miller and Cassady [24] found that nutritional knowledge is important for enhancing individuals’ understanding of dietary modifications and supporting decision-making processes regarding fruit and vegetable consumption. Nutritional knowledge can enrich the ability of construction workers to identify healthy foods from among various food sources, associate nutritional benefits with various food categories, choose proper foods and amounts for their needs based on their health status and disease risk, and to prepare foods in a limited working environment [25,26]. 

Despite the importance of nutritional education for construction workers, studies on training interventions to improve nutritional knowledge or healthy eating among construction workers are limited. One reason for this is that nutritional education programs are expensive, labor-intensive, and involve individual engagement in knowledge dissemination [27]. If nutrition education is to be implemented at worksites, work time must be devoted to training, which is not always appreciated by contractors with whom construction workers do not have long-term employment relationships. Owing to the nature of the work, construction workers are at a disadvantage when it comes to on-the-job training at construction sites. In this respect, one study suggested use of an environmental intervention conducted by placing informational tables in the cafeteria near worksites, to help improve workers’ vegetable consumption [27]. The results showed that the intervention led to significantly increased vegetable consumption among workers, as compared with a control group (*p* < 0.01), although the difference in stages of change was not statistically significant (*p* = 0.05). Although these findings provide some insight into the beneficial effects of passive nutrition knowledge transfer to workers, limitations exist at most worksites in Hong Kong as these do not have a cafeteria in which to purchase food. It is difficult to place such nutritional information at construction sites with catering facilities located in an open area. Therefore, tight working schedules and the lack of a cafeteria are barriers to the implementation of onsite nutritional education programs for employees working at specific building sites [14]. It is also challenging to individualize an environmental intervention to the needs of each worker [27,28]. This could be the reason that environmental interventions do not result in markedly changed eating behaviors.

## 2. Theoretical Framework

According to the transtheoretical model (TTM), stages of change include cognitive and behavioral strategies [28]. Nutrition education focusing on workplace barriers can offer motivational support for nutritional behavioral change, such as increasing vegetable consumption [27]. This can be achieved by improving the relevance of information about healthy foods, rather than only providing access to such information. For instance, instructing construction workers in the skills needed to select affordable, healthy foods, how to easily prepare home-cooked meals, and how to make healthy decisions regarding menu planning is knowledge that can help construction workers to progress from the precontemplation stage to the contemplation, preparation, action, and ultimately the maintenance stage of the TTM. In view of the stages of change, active nutrition education is more effective than passive environmental interventions with respect to healthy nutritional behavior change [20].

A previous study investigated the effect of online nutrition training for construction apprentices [29]. Training topics included the benefits of nutrition, a well-balanced diet, purchasing healthy foods on a budget, nutrition labels, healthier alternatives, healthy packing, snacking, hydration, and food safety. Apprentices demonstrated improved nutrition knowledge after completing the online nutrition training. However, the effects of outcomes indicating behavioral change were small. This makes the effectiveness of online nutrition education questionable in that study population. Nevertheless, those findings shed light on an approach to nutrition training that can be included as part of a skill training course for construction apprentices at training centers. This approach is believed to be feasible because it is easier to set up venues and times according to the working schedule of construction apprentices. Moreover, possessing good nutrition knowledge before commencing job duties can improve apprentices’ understanding of food choices at the workplace and preparedness in making healthy eating choices in the working environment. Because skill training is not conducted at work sites, nutrition education can easily be built into skill training programs for construction apprentices. Therefore, in this study, we aimed to evaluate the effectiveness of a pilot nutrition education program to improve healthy eating knowledge and behavior among construction apprentices. According to the findings of Nagler et al [30], individual approaches to cope with job strain rather than the organizational factors themselves were significantly associated with fruit and vegetable consumption among the construction workers. The effect on food choices could be due to other factors which determine the construction workers’ barriers in consuming fruits and vegetables. Age group which may be related to the construction workers’ understanding in nutrition education and the job strains which may be related to their lack of time in cooking healthy food for themselves. Therefore, individual factors such as age group and working hours could be explored for their effects on the occurrence of a low intake of fruits and vegetables.

Therefore, nutrition education should be designed with respect to a work related context or relevant demographic. The hypothesis of this pilot study was that nutrition education built into construction skill training can enhance construction apprentices’ knowledge and behavior regarding healthy eating, particularly with respect to fruit and vegetable consumption. 

## 3. Methods

The protocol of nutrition education bundled in the skill training courses was approved by the Construction Industry Council. All construction apprentices received nutrition education in their daily training schedule, but they were voluntary to fill in the questionnaires for the research team to conduct data collection and data analysis. Ethics was approved by the Human Research Ethics Committee from the Research and Development Office of the serving university. Written consent was signed by the construction apprentices. 

### 3.1. Design and Setting

This was a pilot evaluative study of a nutrition education program taught as part of standard skill training courses offered to construction apprentices. The training courses were conducted in classrooms at the Hong Kong Institute of Construction, governed by the Construction Industry Council. 

### 3.2. Participants 

Trainees in selected trades under the “Enhanced Construction Manpower Training Scheme” (ECMTS) were invited to pilot the nutrition education program, which was conducted as part of their training schedules. The selected trades under the ECMTS included painting, metal works, metal scaffolding, and timber. These trainees had previously worked in other trades in the construction industry and were attending the full-time ECMTS training programs so as to change job fields. The duration of training programs ranged from 70 days to 94 days, subjected to the type of trades. Two cohorts with a total 36 construction apprentices piloted the proposed nutrition education intervention. There were 20 and 16 participants in the first and second cohorts respectively. We collected demographic characteristics including sex, daily working hours, habits of dining out, and preferred cooking methods using self-administered questionnaires. 

### 3.3. Intervention: Nutrition Education

Two nutrition education sessions (1.5 h each) were conducted for each cohort over two weeks. The sessions were designed to provide nutritional content using pictures, videos, and interactive games to enhance the interest of construction apprentices and motivate them to respond to questions included in the games. Workshops in how to prepare healthy foods were also included to enrich participants’ skills in preparing their own healthy meals. Meals were designed for simple preparation and did not require reheating in order to accommodate construction site settings (Table 1). 

### 3.4. Instrument and Outcome Measures

Because about two-thirds of construction workers in Hong Kong do not receive formal education or complete primary-level education, their literacy levels are low and some have difficulty reading [9]. Therefore, we designed a questionnaire using picture illustrations for the construction apprentices in order to assess their knowledge and behavior regarding healthy eating. In total, there were 12 questions on the survey. Eight questions were used to determine the healthy eating knowledge score (HEKS) and four questions were used to determine the healthy eating behaviour score (HEBS). Responses to these questions comprised categorical data in the analysis. The entire questionnaire was designed by the researcher and reviewed by three scholars in health, nutrition, and building services to ensure that questions related to healthy eating were relevant to construction apprentices’ working environment. The test–retest reliability was 0.84. 

#### 3.4.1. Daily Fruit Consumption and Daily Vegetable Consumption

Fruit and vegetable intake is generally low among construction workers [1]; therefore, enhanced fruit and vegetable consumption in this population was the primary outcome indicator for effectiveness of the nutrition education intervention. The number of daily servings of fruit and vegetables consumed were recorded at baseline, post intervention, and three months after the intervention, to evaluate whether the current mode of nutrition education could promote increased fruit and vegetable consumption among construction apprentices and whether this behavior was maintained at three months after the intervention.

#### 3.4.2. Healthy Eating Knowledge Score (HEKS)

On the survey, a total 17 questions assessed construction apprentices’ knowledge about healthy eating and healthy eating practices in their daily working environment. One question addressed respondents’ understanding of the recommended lunchtime meal, which includes the proper ratio of whole grains, vegetables, and meat (3:2:1). Three questions addressed participants’ judgement about what constitutes a healthy menu, four questions queried knowledge on diet-related diseases, four questions addressed knowledge about healthy cooking methods, three items queried knowledge of low-fat menu options, three questions addressed participants’ understanding of low-sodium menu options, one question queried knowledge of healthy seasonings, one question addressed healthy snacks, and one question queried participants about healthy choices in menu options. One point was given for each correct answer, and these were summed to give the total HEKS. The maximum HEKS was 17. The HEKS was administered at three time points: baseline, post intervention, and three months after the nutrition education intervention.

#### 3.4.3. Healthy Eating Behaviour Score (HEBS)

Three survey questions assessed construction apprentices’ healthy eating behaviors. These included one question about respondents’ consumption of fruit and one question about their consumption of vegetables. One point was given for responses of one serving or less, two points for 2–3 servings, three points for 4–5 servings, and four points for six servings or more. Another item required respondents to pick the healthier food choice between two dishes and correct responses scored one point. All points were summed to give the total HEBS, and the maximum HEBS was 9. The HEBS was administered at three time points: baseline, post intervention, and three months after the nutrition education intervention.

### 3.5. Procedure

Two cohorts of construction apprentices enrolled to the ECMTS were recruited to participate in this study. An information sheet and consent form were provided, and interested apprentices provided a signed consent form. The nutrition education was conducted in the last month of the skill training programs. Demographic and baseline questionnaires, including self-reported daily consumption of fruits and vegetables, pre-intervention HEKS, and pre-intervention HEBS, were distributed to participants for self-administration. After the nutrition education intervention was complete, a post questionnaire (post HEKS and post HEBS) was distributed to participants. Both baseline and post questionnaires were administered to the construction apprentices at the training centres. Three months after the intervention, a follow-up questionnaire (follow-up HEKS and follow-up HEBS) was administered, to assess participants’ healthy eating knowledge and self-reported daily consumption of fruits and vegetables among construction apprentices. By then, the participants returned to work in the construction sites with different trades. A phone number was given to the participating workers and they were requested to administer the follow-up questionnaire at their available time three months after the end of the nutrition education. The participating construction workers were instructed to send the completed questionnaire to the research team by Whatsapp communication.

### 3.6. Data Analysis

IBM SPSS Statistics 26 was used as the data analysis tool in this study. Participants aged 18–40 years and 41–59 years comprised the younger group and older group, respectively. Daily working hours ≤8 h and ≥8 h were categorized as normal working hours and long working hours, respectively. Paired sample t-tests were performed on daily servings of vegetable and fruit consumed at baseline, post intervention, and three months’ follow-up to test the differences in daily fruit and vegetable consumptions at three time points. Enhanced daily fruit and vegetables consumption at post intervention as compared with those at baseline demonstrated the effectiveness of the proposed intervention and enhanced daily fruit and vegetables consumption at three months’ follow-up demonstrated sustainability of the proposed intervention. Paired sample t-tests were also performed on HEKS and HEBS at the three time points to test any differences resulted after the intervention. Linear regressions were conducted to investigate differences of effects between age groups and between working hours in the HEKS and HEBS at the corresponding three time points. Significance levels were all set to α = 0.05. Bonferroni adjustment was also used in the analysis to control possible inflation of Type I error in multiple comparisons (HEKS, HKBS, three time points, two age groups, and two working hours groups) of significance [31].

## 4. Results

Two cohorts of trainees receiving full-time training courses in operative and craft skills were recruited between June and November 2018. The training programs included three hours of nutrition education as part of construction training, with two sessions of 1.5 hours each, given one week apart. Participants comprised 36 construction apprentices in total. Among them, 86.1% (*n* = 31) were male and 13.9% (*n* = 5) were female apprentices. About two third participants were age 18–40 years and one-third were age 41–59 years. Around 27.8% of construction apprentices worked less than eight hours daily and two-thirds of them worked more than eight hours daily. The ratio of eating at home to dining out was 2:1. The most preferred cooking methods included pan frying (*n* = 27, 75%), stir frying (*n* = 27, 75%), and steaming (*n* = 19, 52.8%) (Table 2). 

### 4.1. Effect of Intervention on Fruit and Vegetable Consumption

Results found that the baseline means daily fruit consumption and daily vegetable consumption were 1.42 servings and 1.67 servings which were lower than the recommended guideline [6]. The number of servings for fruit and vegetable, however, increased significantly after the nutrition education implemented in the skill program (*p* < 0.05). After three months, the amount of consumption of fruit and vegetable further increased, though not in significant amounts as compared with the number of servings reported at the post intervention (*p* > 0.05). 

### 4.2. Effect of Intervention on Healthy Eating Knowledge and Behaviour

With respect to healthy eating knowledge, the mean baseline HEKS was 10.8. Compared with the maximum score of 17, the mean baseline HEKS scores were moderate as far as demonstrating knowledge about healthy eating in participants’ occupational environment. Results showed that nutrition education did not improve the healthy eating knowledge of construction apprentices immediately after the intervention, but it improved the follow up healthy eating knowledge significantly (*p* < 0.05). The mean baseline HEBS was 3.83. Compared with the maximum score of 9, mean baseline HEBS results were also unsatisfactory. The proposed nutrition education however improved the healthy eating behaviour of the participated apprentices significantly from baseline to post intervention (*p* < 0.05) and from post intervention to three months after the intervention (*p* <0.05) (Table 3). Pearson correlation coefficients between baseline HEKS and baseline HEBS, post HEKS and post HEBS and follow-up HEKS and follow-up HEBS were *r* = 0.131 (*p* > 0.05), *r* = 0.111 (*p* > 0.05) and *r* = −0.301 (*p* > 0.05) respectively.

### 4.3. Effect of Intervention on Healthy Eating Knowledge by Age Group and Working Hours

For every one unit increase in age group (from younger group to older group), the HEKS increases by 0.993. This implied older construction apprentices had similar HEKS to the younger apprentices before the nutrition education. For every one unit increase in working hours (from group of working in normal hours to group of working in longer hours), the HEKS increases by 0.762. This implied construction apprentices working in longer hours had lower HEKS than those working in normal hours before the nutrition education. The relationships between age group or working hours and the baseline HEKS were not significant (Table 4).

After the nutrition education, younger age group and those working in normal time duration were found having better HEKS than their counterparts. From the younger group to the older group, the HEKS decreased by 1.552, and from working normal hours to working beyond normal hours the HEKS decreased by 1.631. The relationships between age group or working hours and the post HEKS again were not significant (Table 4).

After three months of the intervention, younger group and those working in normal time duration were found having better HEKS. From younger group to older group, the HEKS decreased by 1.217 and from working normal hours to working beyond normal hours, the HEKS decreased by 0.169. The effect of working hours on mean follow-up HEKS was small. The relationship between age group or working hours and the follow-up HEKS, again were not significant (Table 4). 

### 4.4. Effect of Intervention on Healthy Eating Behaviour by Age Group and Working Hours

For every one unit increase in age group (from younger group to older group), the HEBS increases by 0.502. This implied older construction apprentices had lower HEBS than the younger apprentices before the nutrition education. For every one unit increase in working hours (from group of working in normal hours to group of working in longer hours), the HEBS decreased by 0.508. This implied that construction apprentices working longer hours had lower HEBS before the nutrition education. The relationships between age group or working hours and the baseline HEBS were not significant (Table 4).

After the nutrition education, younger age group and those working in normal time duration were found having better HEBS than their counterparts. From the younger group to the older group, the HEBS increased by 0.368 and from working normal hours to working beyond normal hours, the HEBS decreased by 0.262. The effect of age group and working hours on mean follow-up HEKS were small. The relationships between age group or working hours and the post HEBS again were not significant (Table 4).

After three months of the intervention, younger group and those working in normal time duration were found having better HEBS. From younger group to older group, the HEBS decreased by 0.227 and from working normal hours to working beyond normal hours, the HEBS increased by 0.585. The effect of age group on mean follow-up HEBS was small. The relationship between age group or working hours and the follow-up HEBS, again were not significant (Table 4). 

## 5. Discussion

The International Labour Organization (ILO) issued a statement regarding good nutrition as it is related to health and workplace productivity [32]. Good nutrition determines the concentration and alertness of workers, which in turn reduces the risks of accidents, injuries, and fatal events [33]. Accompanied by the problem of an aging workforce in the construction industry, poor nutrition accounts for about one-third of the deterioration in physical work capacity and one-fifth the loss in worker productivity [18]. Less healthy cooking methods, such as pan frying and stir frying, accounted for the top two preferences among construction apprentices in our study. Results from the baseline questionnaire, the percentage of our participants who did not consume the recommended intake of vegetables and fruit was high, and more participants had low fruit consumption than low vegetable consumption. This indicates that the construction apprentices were less aware about healthy eating and were thus more vulnerable to the risk of diet-related diseases and poorer health. This finding also revealed that an adequate intake of fruit and vegetables was frequently neglected among construction apprentices. In fact, workers in the construction industries are found experiencing structural barriers in having fruit and vegetables in the recommended amount. A previous study identified that construction workers, and especially male workers, have less knowledge about food choices. This population believes that consuming high-fat foods helps them to have more energy to perform physically demanding tasks [34]. This misunderstanding about food choices could be associated with the high prevalence of elevated cholesterol concentrations (38.4%) and hypertension (39.8%) in the construction workforce of Hong Kong [12]. 

### 5.1. Effect of Intervention on Daily Fruit and Vegetable Consumptions

The participated construction apprentices increased both daily fruit consumption and daily vegetable consumption significantly, demonstrating an effective strategy by using skill training as an opportunity in promoting fruit and vegetable consumption. Although the number of servings still cannot meet the recommended intake, the effects were effective, taking account of a minimal time spending on the lessons (three hours), contributing 0.4%–0.5% of total learning hours of the skill training program. The effects of the nutrition education on fruit and vegetable consumptions was shown effective immediately after the intervention. Though the increase of fruit and vegetable consumption from post intervention to follow up was not significant, the intervention effect was sustained at three months after intervention. The findings suggested the effect of healthy food choices was sustained because the mean daily intake of fruits and vegetables increased further and no significant drop in consumption at three months. This indicated the education demonstrate a markable impact to influence workers’ food choice. The findings of this study supported previous research that individualized nutrition programs were necessary to help construction workers to consume more fruit and vegetables [30]. 

### 5.2. Effect of Intervention on Healthy Eating Knowledge and Behaviour

The significant increase in healthy eating knowledge happened after 3 months, and thus not immediately. This was possibly an indication of construction apprentices’ ability to acquire nutrition knowledge after the intervention. It is evident that nutrition knowledge is determined by education level [35] and construction apprentices are usually at lower education level. Therefore the construction apprentices may need more time to understand and apply nutrition knowledge by repeating and refreshing practices in their daily life. The significant increase of HEKS after three months may possibly be explained by the addition awareness of nutritional requirements for their health after attending the nutrition education. For the significant increase of HEBS at both post intervention and three months follow up, Okoro et al. [36] established substantial analysis that indicates an understanding of alternative foods enhances dietary modifications and promotes positive healthy eating behaviour. To this, healthy workshop in the nutrition education may help the construction apprentices to remove barriers of healthy eating, menu analysis in local restaurants designed in the education content may help the construction apprentices to modify their eating behaviour and support their continual healthy eating behaviour. 

### 5.3. Relationship of Age Group on Healthy Eating Knowledge and Behaviour

The findings of Deacon and Smallwood [37] indicate that around 40% of construction contractors are unaware about or disagree with the benefits of nutrition for their health and safety. With such limited awareness about healthy eating, nutritional knowledge is suggested as a prerequisite for making changes in eating behaviors [24,38]. At the three time points measured in this study, the daily vegetable consumption and daily fruit consumption of construction apprentices was increased immediately after the nutrition education intervention and further increased at three months of follow-up. A difference of HEKS was shown in age groups at post intervention and three months follow-up. This revealed that effective knowledge transfer with the implementation of three-hour nutrition education in a skill training program may be better in the young age group. The maintenance of healthy eating knowledge may also be better in the young age group. The knowledge transfer effect on the older group may probably be enhanced by having more than three hours of nutrition education. The cost-effectiveness of intervention design however requires further research to support the effects of this learning strategy among the construction workers.

Regarding the overall healthy eating behaviour, our results reflected participants’ age may not influence their eating habits on healthy eating. The magnitude in differences of healthy eating behavior was not aligned with those of healthy eating knowledge, indicating the enhanced nutrition knowledge may not transfer to practice in healthy eating. In contrast with the results of enhancement in daily intake of fruit and vegetable. This implied the nutrition education was possibly effective to the daily fruit and vegetable consumption specifically, not the overall healthy eating behavior. As fruit and vegetable intake is only a part of overall healthy eating habits, this proposed nutrition education may promote the fruit and vegetable consumption as set but not as effective to promote healthy eating in overall food choice in proper amount.

### 5.4. Relationship of Daily Working Hours and Healthy Eating Knowledge and Behaviour

Healthy eating knowledge and healthy eating behaviour were shown to have no differences between participants working normal hours and beyond normal hours. In fact, healthy eating knowledge and behaviour promoted one another [39]. Although healthy eating knowledge and healthy eating behaviour were not highly correlated in this study, there could be a confounding factor that may lead to the change. For example, health status may make some construction workers more aware on their healthy eating practice and they may pay more attention on their diet after the nutrition education. The practice of healthy eating applied the healthy eating knowledge gained in nutrition education. The results in this study showed that those working longer hours had lower healthy eating knowledge than those working in normal hour. Construction workers with longer working hours may find it difficult to practice healthy eating as the learned knowledge could not be reviewed and applied after the nutrition education. The effects of differences in working hours on HEKS at follow up became smaller which indicated the healthy eating knowledge between those two groups was similar, though the mean HEKS increased. Therefore, continued practice of healthy eating may support the learning of healthy eating knowledge across the time.

The differences of HEBS between the normal and longer hours working groups at all time points were small. The increasing HEBS found at post nutrition education and three months after may not be affected by the daily working hours. The nutrition education designed with construction context could be useful to help the construction apprentices transferring healthy eating knowledge to healthy eating behaviour, regardless of their daily working hours.

### 5.5. Limitation

This study included several limitations that may need cautious attention to address the intervention effects. First, it was a pilot study with small sample size, the validity of which could be examined in a further study. Second, the control group was not included in the research design and possible demographic factors were not controlled for by covariate analysis. However, the findings constitute a foundation for the design of intervention programs to promote healthy eating among construction workers at the training stage.

## 6. Conclusions

Our results support that despite implementing nutrition education as only a 3-h session within construction skills courses, the intervention had some extent of increase in daily fruit consumption and daily vegetable consumption. The impact of nutrition education on healthy eating knowledge and behaviors among construction apprentices were not significantly different between age groups or working hours. Significant increases in healthy eating knowledge at three months follow up and healthy eating behaviour were observed in apprentices at the post intervention and three months follow up, yet further studies are needed to support the findings. These findings highlighted the possible strategies in transferring nutrition knowledge to construction apprentices or workers. The findings also provided some insights into the difference of effectiveness of three hours nutrition education between young and old construction workers in that older construction workers may require a longer time to develop healthy eating knowledge.

## Figures and Tables

**Table 1:** Topics and content of nutrition education for construction apprentices.

Topic	Content	TTM Stage †
Relationship of diet and diseases	Diet-related diseases with the related nutrients deficiencies and nutrients in excess were introduced.	Precontemplation stage: to facilitate the participants aware of nutrients of food affect the risk of chronic diseases and health.
Nutrients for healthy eating	Macronutrients and micronutrients in food were introduced with functions and benefits to health.	Contemplation stage: to increase the understanding of nutrients of food in health maintenance.
Balanced diet	Balanced diet defined as eating different categories of food in recommended serving size and number of servings. Examples of food in each category. Processed food items frequently mistakenly perceived as healthy foods were addressed.	Contemplation stage: to increase the understanding of food categories in balanced diet.
Menu in local restaurants	Food analysis on common dishes provided by local restaurants. Healthy choices of dishes from some collected menu were discussed.	Preparation stage: to apply healthy eating knowledge in daily lives.
Healthy food choices	Healthy food choices in supermarkets and restaurants.	Preparation stage: to purchase healthy ingredients for cooking, not the processed food for cooking meals.
Healthy cooking workshop	Healthy cooking and unhealthy cooking. Use of sauce, gravy, hydrogenated oils and herbs was discussed. Meals were designed for simple preparation and did not require reheating, to accommodate construction site settings.	Action stage: to enrich participants’ skills in preparing their own healthy meals.
Daily food records for dietary monitoring	Construction workers were advised to record their food intake for dietary monitoring after the nutrition education, at voluntary basis.	Maintenance stage: to encourage participants to keep attention on their food choice and portion size for the promotion of fruit and vegetable consumption.

† TTM: transtheoretical model includes stages of precontemplation, contemplation, preparation, action and maintenance.

**Table 2:** Characteristics of participating construction workers.

*N* = 36	*n* (%)
Sex	
Male	31 (86.1)
Female	5 (13.9)
Age group	
18–40	23 (63.9)
41–59	13 (36.1)
Daily working hours	
≤8	10 (27.8)
>8	26 (72.3)
Eating location	
Eating out	12 (33.3)
Eating at home	23 (63.9)
Preferred cooking method *	
Pan fried	27 (75.0)
Stir fried	27 (75.0)
Steamed	19 (52.8)
Deep fried	15 (41.7)
Simmered	15 (41.7)
Stewed	14 (38.9)
Roasted	13 (36.1)
Boiled	11 (30.6)
Marinated	7 (19.4)

* Multiple options allowed.

**Table 3:** Summary of t-tests of HEKS and HEBS.

Outcome Measures	Mean (s.d.)	95% Confidence Interval	T
Daily fruit consumption (serving)			
Baseline	1.42 (0.55)		
Post nutrition education	1.72 (0.70)	(−0.595, −0.016)	−2.142 (*p* < 0.05)
Follow up	1.94 (0.83)	(−0.537, 0.092)	−1.435 (*p* > 0.05)
Daily vegetable consumption (serving)			
Baseline	1.67 (0.59)		
Post nutrition education	1.97 (0.74)	(−0.584, −0.027)	−2.231 (*p* < 0.05)
Follow up	2.19 (0.82)	(−0.492, 0.047)	−1.673 (*p* > 0.05)
HEKS			
Baseline	10.8 (3.12)		
Post nutrition education	11.2 (3.30)	(−1.568, 0.623))	− 0.875 (*p* > 0.05)
Follow up	14.8 (2.37)	(−4.939, −2.172)	−5.219 (*p* < 0.05)
HEBS			
Baseline	3.83 (1.08)		
Post nutrition education	4.61 (1.50)	(−1.270, −0.285)	−3.205 (*p* < 0.05)
Follow up	5.22 (1.85)	(−1.179, −0.043)	−2.185 (*p* < 0.05)

HEKS: Healthy Eating Knowledge Score; HEBS: Healthy Eating Behaviour Score.

**Table 4:** Linear regression of age group and working hours on HEKS and HEBS.

Source	*B*	*SEB*	*β*	*T*	*P*
HEKS					
baseline					
Age group	0.993	1.085	0.155	0.915	0.366
Working hours	0.762	1.171	0.111	0.650	0.520
Post nutrition education					
Age group	−1.552	1.129	−0.229	−1.374	0.178
Working hours	−1.631	1.213	−0.225	−1.345	0.188
Follow up					
Age group	−1.217	0.807	−0.250	−1.509	0.141
Working hours	−0.169	0.893	−0.032	−0.189	0.851
HKBS					
baseline					
Age group	0.502	0.371	0.226	1.351	0.185
Working hours	−0.508	0.399	−0.213	−1.272	0.212
Post nutrition education					
Age group	0.368	0.524	0.120	0.703	0.487
Working hours	−0.262	0.564	−0.079	−0.464	0.646
Follow up					
Age group	−0.227	0.651	−0.060	−0.349	0.729
Working hours	0.585	0.692	0.143	0.844	0.404

*B*: the unstandardized beta; *SEB*: the standard error for the unstandardized beta; *β*: the standardized beta; *p*: the probability value; *HEKS*: healthy eating knowledge score; *HEBS*: healthy eating behaviour score.
